# Multicomponent Peptide Hydrogels as an Innovative Platform for Cell-Based Tissue Engineering in the Dental Pulp

**DOI:** 10.3390/pharmaceutics13101575

**Published:** 2021-09-28

**Authors:** Marina E. Afami, Ikhlas El Karim, Imad About, Anna D. Krasnodembskaya, Garry Laverty, Fionnuala T. Lundy

**Affiliations:** 1 Wellcome-Wolfson Institute for Experimental Medicine, School of Medicine, Dentistry and Biomedical Sciences, Queen’s University Belfast, 97 Lisburn Road, Belfast BT9 7BL, UK; mafami01@qub.ac.uk (M.E.A.); i.elkarim@qub.ac.uk (I.E.K.); a.krasnodembskaya@qub.ac.uk (A.D.K.); 2 Aix Marseille Univ, CNRS, ISM, Inst Movement Sci, 13385 Marseille, France; imad.about@univ-amu.fr; 3 School of Pharmacy, Queen’s University Belfast, 97 Lisburn Road, Belfast BT9 7BL, UK; garry.laverty@qub.ac.uk

**Keywords:** antibacterial, antimicrobial, biocompatibility, biofilm, cytokine, dental pulp, growth factor, hydrogel, oral pathogen, secretome

## Abstract

In light of the increasing levels of antibiotic resistance, nanomaterials and novel biologics are urgently required to manage bacterial infections. To date, commercially available self-assembling peptide hydrogels have not been studied extensively for their ability to inhibit micro-organisms relevant to tissue engineering sites such as dental root canals. In this work, we assess the biocompatibility of dental pulp stem/stromal cells with commercially available multicomponent peptide hydrogels. We also determine the effects of dental pulp stem/stromal cell (DPSC) culture in hydrogels on growth factor/cytokine expression. Furthermore, to investigate novel aspects of self-assembling peptide hydrogels, we determine their antimicrobial activity against the oral pathogens *Staphylococcus aureus*, *Enterococcus faecalis*, and *Fusobacterium nucleatum*. We show that self-assembling peptide hydrogels and hydrogels functionalized with the adhesion motif Arg-Gly-Asp (RGD) are biocompatible with DPSCs, and that cells grown in 3D hydrogel cultures produce a discrete secretome compared with 2D-cultured cells. Furthermore, we show that soluble peptides and assembled hydrogels have antimicrobial effects against oral pathogens. Given their antibacterial activity against oral pathogens, biocompatibility with dental pulp stem/stromal cells and enhancement of an angiogenic secretome, multicomponent peptide hydrogels hold promise for translational use.

## 1. Introduction

The oral cavity harbours over 700 distinct microbial species that occupy several niches in the oral cavity, including the hard surfaces of the teeth, and the mucosal surfaces of the gums, cheeks and tongue [1]. The non-shedding surfaces of the teeth facilitate the accumulation of microorganisms in the form of dental plaque, which in the absence of good dietary and oral hygiene habits can lead to the development of dental caries. If left untreated, dental caries will extend through the enamel and dentin layers to reach the dental pulp, causing pain and inflammation. Teeth in which the dental pulp has become infected following ingress of caries would conventionally have been treated by root canal treatment, in which the entire dental pulp is removed. However, there is now much interest in dental pulp regeneration, in which scaffolds/nanomaterials are used to deliver stem cells that are either transplanted (allogenic cells) or attracted to the site (autologous cells). For tissue engineering approaches to be successful, there should be effective eradication of infection from the root canals of the teeth; otherwise, there is a high risk of treatment failure [2]. The requirement for effective disinfection in regenerative procedures is possibly more stringent than with conventional endodontic therapies [2], such as standard root canal treatments, which are well known to have a higher risk of failure if disinfection is incomplete. Although many bacterial taxa have been identified in root canals, the current study focuses on three bacteria that have been documented to play particularly important roles in endodontic infection. *Enterococcus faecalis* has been reported to have a major role in the aetiology of persistent infections and is able to survive in the root canal as a single organism or as a major component of the microbiota [3,4]. Several studies have also detected high rates of the Gram-negative anaerobic rod, *Fusobacterium nucleatum,* in infected root canals. The presence of *F. nucleatum* has been reported in teeth with both primary infection [5] and persistent endodontic infection [6]. *Staphylococcus aureus* is another microorganism commonly found in infected root canals [7] and is one of the major causes for the failure of standard root canal treatment [8].

Hydrogels (water-swollen networks of polymers) have emerged as particularly attractive materials for biomedical and dental applications because of their ability to mimic the in vivo extracellular environment in three dimensions [9]. Hydrogels have been shown to support cell adhesion and demonstrate biocompatible properties [9]. Furthermore, they can be specifically designed to have mechanical properties resembling those of tissues in vivo. Despite the urgent need for novel biologics to manage bacterial infections in the oral cavity and other bodily sites, much remains to be learned about the development of hydrogels with novel antimicrobial properties. Amongst the many classes of hydrogels in current use (containing synthetic polymers or natural polymers), peptide hydrogels are a promising class. Their chemical versatility, providing a vast choice of chemical functional groups, enables tailoring of the peptide primary sequence to required functional properties (e.g., mechanical properties, syringeability) for specific application.

Peptides are ubiquitous in nature, and the sequence of their amino acids specifically determines their biological functions, including their propensity to be antimicrobial [10]. Peptide hydrogels that self-assemble are often categorised as hybrid hydrogels, as they retain the biocompatibility of naturally derived extracellular matrix molecules, but are synthesised using standard peptide synthesis techniques [11]. Peptide hydrogels bridge the gap between the use of naturally occurring molecules and chemically synthesised molecules as hydrogel polymers. Self-assembling peptide hydrogels are thus smart/designer novel biologics with a wide range of potential therapeutic applications. Amongst their key attributes such as their biocompatibility and ease of synthesis, antimicrobial activity is emerging as a clinically relevant novel characteristic that is worthy of exploitation for the management of bacterial infections. Indeed, animal models have shown that residual bacteria limit tissue mineralization in regenerative endodontic procedures [12]. Although hydrogels are used experimentally in regenerative endodontics, the majority of current research is focused on the development of hydrogels that are biocompatible with cells in the local environment. Whilst this is of course an essential attribute for hydrogels designed for tissue engineering purposes, it is only one factor that contributes to the development of a successful novel biologic. Our research group is interested in the application of biocompatible hydrogels with inherent antimicrobial characteristics for use in tissue engineering [13] in the oral cavity. We previously demonstrated the antibacterial properties associated with ‘in-house’ ultra-short peptide hydrogels [13,14] specifically designed to exhibit antimicrobial activity against oral pathogens [13], and now seek to address the possibility that commercially available multicomponent peptide hydrogels may also exhibit this favourable characteristic.

Peptide hydrogels functionalized with the cell adhesion motif arginine-glycine-aspartic acid (RGD) have the components necessary to promote self-assembly, along with the cell adhesive properties of RGD, which is well documented as a recognition site and anchor for cell adhesion [15]. Indeed, recent work has shown that the RGD motif enhances cell–matrix interactions and promotes cell survival/proliferation [16] in functionalized hyaluroic acid hydrogels. Further investigations are needed to determine the role of RGD in multicomponent hydrogels and expedite their translation to clinical use. We therefore hypothesised that commercially available multicomponent peptide hydrogels (+/− RGD adhesion motifs) may have unrecognised antimicrobial activity, as this characteristic of peptide hydrogels has generally not been tested, except for hydrogels specifically designed for this purpose. There have been no previous reports on the influence of the RGD motif on antimicrobial activity, but given its important role in cellular adhesion and survival/proliferation, we sought to determine whether it influenced antimicrobial activity in multicomponent hydrogels. Herein, we studied commercially available peptide hydrogels and custom synthesised RGD-containing peptide hydrogels, with a view to demonstrating their biocompatibility with dental pulp cells, their influence on the secretome of dental pulp cells and their propensity for antimicrobial activity against the oral pathogens *E. faecalis*, *F. nucleatum* and *S. aureus*.

## 2. Materials and Methods

### 2.1. Peptide Hydrogels

Peptide hydrogels (Biogelx^TM^-S or Biogelx^TM^-RGD) were manufactured by Biogelx (Newhouse, Scotland). The Biogelx^TM^-S formulation, containing 50% 9-fluorenylmethoxycarbonyl-diphenylalanine (Fmoc-FF) and 50% 9-fluorenylmethoxycarbonyl-serine (Fmoc-S), is a standard hydrogel formulation without adhesion motifs, and is subsequently referred to as 0% RGD. The RGD containing peptide hydrogels (Biogelx^TM^-RGD) also contained 9-fluorenylmethoxycarbonyl-RGD (Fmoc-RGD) and were custom synthesised (Biogelx, Newhouse, Scotland) with various percentages of RGD-functionalized peptides (10%, 25%, 40%, 50% RGD), as detailed in Table 1. All peptides were assembled in hydrogel form as described by the manufacturer. Briefly, the weight of the powder dissolved in water was adjusted according to desired gel volume and stiffness. All assembled hydrogels in this study were prepared at concentrations of 4.4–5.4 mg/mL, according to the manufacturer’s instructions. These concentrations of peptides were described by the manufacturer to exhibit rigidity within the range of 0.8–1.1 kPa, when assembled in hydrogel form. The solution of peptides in water was stored overnight at 4 °C to allow stabilization of the fibres, prior to the initiation of gelation the following day, upon addition of cell culture media. Peptides in assembled hydrogel form were utilised in modified 3-(4,5-dimethylthiazol-2-yl)-2,5-diphenyltetrazolium bromide (MTT) assays [17], cell encapsulation studies, secretome studies and bacterial susceptibility assays. In addition, peptides in their soluble form were investigated for their biocompatibility with a sub-population of dental mesenchymal stem cells, which were positive for the mesenchymal marker, stromal precursor antigen 1 (Stro-1). Peptides in solution form were also tested for their antibiofilm properties. Peptides were maintained in solution form by dissolving the peptides in culture media containing 0.005% DMSO (Sigma-Aldrich, Dorset, UK).

### 2.2. Explant Culture of Dental Pulp Cells

Samples of human dental pulp tissue [18] were obtained in accordance with French ethics legislation and approved by the Office for Research Ethics Committees (Northern Ireland) ethical approval number 08/NIR03/15, dated 11/04/08. The dental pulp tissue was cut into small fragments using a sharp scalpel and cultured using the previously described explant method [19]. Cells were cultured in *α*-MEM (Thermo Fisher Scientific, Waltham, MA, USA), supplemented with 10% heat inactivated foetal bovine serum (Thermo Fisher Scientific, Waltham, MA, USA), 1% *w*/*v* L-glutamine (L-glut) (Thermo Fisher Scientific, Waltham, MA, USA) and 1% *w*/*v* penicillin + streptomycin (Thermo Fisher Scientific, Waltham, MA, USA). Dental pulp cells, often collectively referred to as dental pulp stem/stromal cells (DPSCs), were grown at 37 °C with 5% CO_2_, to a confluence of approximately 80%. Cells of passages 2–4 were used throughout all experiments.

### 2.3. Biocompatibility Screening of Peptides in 2D Culture

The cytotoxicity of a series of multicomponent peptide hydrogels (0%, 10%, 25%, 40% and 50% RGD) was initially screened in their soluble forms against a population of Stro-1-positive DPSCs using a standard MTT assay. In the absence of a technique that results in a perfectly homogenous population of mesenchymal stem cells [20], Stro-1 is often used as a marker of multipotent mesenchymal progenitor cells in the dental pulp. Stro-1-positive cells were obtained as previously described by us using immunomagentic sorting [21]. The Stro-1-positive DPSCs (100 µL; 1 × 10^4^ cells/mL) were transferred to each well of a flat-bottomed 96-well microtitre plate (Nunclon Delta Surface, Thermo Fisher Scientific, Waltham, MA, USA) and incubated for 48 h at 37 °C in a humidified atmosphere of 5% CO_2_. After 48 h incubation, the medium was aspirated from the wells and replaced with 100 µL of liquid peptides at concentrations of 0.01% and 0.1% *w*/*v* for 24 or 72 h. When hydrogels are formed, it is recognised that small proportions of the peptides may not incorporate into the gel but instead diffuse into the solution [14]. As a result, soluble peptides may be present on the surface of the hydrogel. Greater than 0.44% *w*/*v* peptide concentrations were required for gelation to occur in all five (0%, 10%, 25%, 40% or 50% RGD) hydrogels tested (according to the manufacturer’s instructions). We therefore prepared 0.1% *w*/*v* and 0.01% *w*/*v* solutions of soluble peptides (as outlined in Section 2.1) to determine their potential cytotoxicity at two different concentrations below the critical gelation concentration for each hydrogel. This was undertaken in order to test the potential cell cytotoxicity of material extracts in line with International Standard (ISO 10993-5) methods for biomaterials. In addition to treating cells with 0.1% *w*/*v* and 0.01% *w*/*v* peptides, the control wells contained 100 µL of media per well in place of the soluble peptides. After 24 or 72 h, 10 µL of MTT (5 mg/mL in HBSS) (Sigma-Aldrich, Dorset, UK) was added to the wells [22] and incubated for 3 h. Following this, the medium was removed and the wells were allowed to air dry for 10 min. Formazan crystals, representing mitochondrial activity, were dissolved in 200 µL DMSO (Sigma-Aldrich, Dorset, UK) and the absorbance was measured at 510 nm using a plate reader (Genios Spectrophotometer). Stro-1-positive DPSCs were employed only for initial screening purposes, and mixed population DPSCs (subsequently referred to as DPSCs) were studied in the remaining experiments.

### 2.4. DPSC Encapsulation in Assembled Hydrogels

DPSCs (1 × 10^6^ cells/mL) were encapsulated in multicomponent peptide hydrogels containing 0%, 10%, 25%, 40% and 50% RGD (100 µL of cells were encapsulated in 100 µL of hydrogel) and plated in glass bottom dishes (WillCo Wells BV, Amsterdam, The Netherlands). Before plating the encapsulated cells, an area on the bottom of each dish was marked with a hydrophobic barrier pen (Fisher Scientific, Dublin, Ireland) to ensure that the hydrogels were retained within a defined area. Then, 200 µL *α*-MEM was gently added on top, and the cells were incubated in the 3D hydrogel construct for 24 h. Cells were then fixed in 4% paraformaldehyde for 30 min and washed three times with phosphate buffered saline (PBS) before adding several drops of ProLong^TM^ Gold Antifade Mountant with 4′,6-diamidino-2-phenylindole (DAPI; ThermoFisher Scientific, Warrington, UK). The hydrogel construct was then cured at room temperature for 24 h prior to the visualisation of encapsulated cells by SP8 Confocal Microscopy (Leica, Wetzlar, Germany). Z stack images were acquired and processed using LAS X software (Leica Microsystems, Wetzlar, Germany).

### 2.5. Biocompatibility of Assembled Hydrogels with DPSCs in 3D Culture

DPSCs (1 × 10^6^ cells/mL) were encapsulated in hydrogels (0%, 10%, 25%, 40% and 50% RGD) and cultured in 200 µL *α*-MEM as outlined above. A modified MTT assay for 3D culture was employed to determine DPSC viability [23]. Cells cultured in 2% *w*/*v* hydroxypropyl methylcellulose (HPMC) hydrogels were used as controls. The viability of DPSCs was determined at 1 and 14 days following cell encapsulation. Briefly, 20 µL of MTT (5 mg/mL in Hanks balanced salt solution) was added to each well and incubated for 1 h at 37 °C. The cell medium (200 µL) was then carefully removed and replaced with DMSO (200 µL) and incubated at 37 °C for 10 min. The blue MTT–formazan product was then eluted by shaking the plate at 220 rpm for 3 h. Following dye elution, the supernatant from each well was transferred to a new plate and the absorbance measured at 570 nm on a microplate reader (Thermo Scientific Varioskan LUX, Warrington, UK) using SkanIt RE 4.1 software.

### 2.6. Antibacterial Activity Assays

The antibacterial activities of 0% RGD and 10% RGD peptides in both solution and assembled hydrogel form were tested. The antibacterial effects of low concentrations (0.01% and 0.1% *w*/*v*) of peptides in solution (representing soluble peptides that may diffuse from the hydrogel surface [14,24]) were tested against bacterial biofilms in 96-well microtitre plates. Prior to establishing biofilms, *S. aureus* (ATCC 25923) and *E. faecalis* (NCTC 12697) were grown aerobically in Müeller–Hinton broth and the broth was allowed to reach mid-Logarithmic phase (6 h), before dilution to 1 × 10^6^ colony forming units (CFU)/mL in Müeller–Hinton broth to obtain the desired inoculums for the biofilm assay. The inoculums (100 µL per well) were incubated aerobically for 5 h to establish initial *S. aureus* and *E. faecalis* biofilms. *F. nucleatum* (NCTC 10562) was grown anaerobically in fastidious anaerobe broth (LabM Limited, Lancashire, UK). On reaching the mid-logarithmic phase (15 h), it was diluted to 5 × 10^6^ CFU/mL in anaerobic broth to obtain the desired inoculum for the biofilm assay. Wells containing 100 µL of inoculum were incubated anaerobically for 48 h to establish initial *F. nucleatum* biofilms.

Following the establishment of initial biofilms, planktonic bacteria were removed by carefully washing with PBS, and the biofilms were treated with 100 µL of 0% RGD or 10% RGD in solution form at concentrations of 0.01% and 0.1% *w*/*v*. Control wells for each micro-organism were treated with the vehicle only (broth containing 0.005% DMSO). Following treatment, biofilms were allowed to mature either aerobically for a further 24 h (*S. aureus* and *E. faecalis*) or anaerobically for a further 48 h (*F. nucleatum*). After incubation for either 24 or 48 h, planktonic bacteria were removed by washing and the biofilm biomass was quantified by the crystal violet assay [25].

To test whether the assembled hydrogels had antibacterial activity, bacterial viability was determined by the colony count method [26]. Briefly, inoculums of *S. aureus* and *E. faecalis* (2 × 10^6^ CFU/mL) or *F. nucleatum* (5 × 10^6^ CFU/mL) were prepared as outlined above. Bacterial suspensions (100 µL) were seeded on top of 100 µL 0% RGD or 10% RGD assembled hydrogels in 96-well plates. Bacterial suspensions (100 µL) served as untreated control wells. After incubation for 24 h, a sample of the bacterial suspension (20 µL) was removed from each well and serially diluted. A total volume of 10 µL from each dilution was plated out on appropriate agar plates for viability counting. The results were displayed as mean Log_10_ CFU/mL.

### 2.7. Human Growth Factor/Cytokine Array

Human growth factor/cytokine array membranes were purchased from Abcam (Cambridge UK) for the detection and semi-quantification of 43 growth factors/cytokines in the secretome of DPSCs following 2D or 3D culture. Conditioned medium was collected on day 14, and the assay procedure was performed as described in the manufacturer’s instructions. The arrays were imaged using a Syngene G: BOX, with GeneSys software and semiquantified using ImageJ, an image analysis programme, with a microarray plugin. For each array membrane, the integrated density of the spots was measured and then the mean integrated density of negative controls was subtracted for background correction. To ensure that array results could be compared across the various experimental conditions (2D versus 0% RGD or 10% RGD), the 2D array was designated as the reference array membrane. The positive control spots on the 2D reference array were then used to normalise the signal intensity (integrated density) on the 0% RGD and 10% RGD membranes. The normalised integrated density for each growth factor/cytokine on any given array was calculated using the formula:

*X*(*Ny*) = *X*(*y*) × *P1*/*P*(*y*)

where: *X*(*Ny*) = Normalized signal density for spot “*X*” on array “*y*”

*X*(*y*) = Signal density for spot “*X*” on array “*y*”

*P1* = Mean signal density of positive control spots on reference array (2D array)

*P*(*y*) = Mean signal density of positive control spots on array “*y*” (0% RGD or 10% RGD arrays).

### 2.8. Statistical Analysis

All results were expressed as the mean of three independent experiments with three to six replicates per experiment. Statistical analysis was undertaken using GraphPad Prism Software (Version 8; GraphPad Software Inc., San Diego, CA, USA). Data that passed the Shapiro–Wilk or D’agostino and Pearson test were analysed by means of one-way ANOVA followed by Dunnett’s post hoc correction for multiple comparisons. Otherwise, data were analysed non-parametrically by means of a Kruskal–Wallis test with Dunn’s correction for multiple comparison testing, as indicated in the figure legends. The level of statistical significance was set at *p* < 0.05.

## 3. Results

### 3.1. Biocompatibility of Soluble Peptides and Assembled Hydrogels (+/− RGD Functionalization) with DPSCs

**Figure 1:**
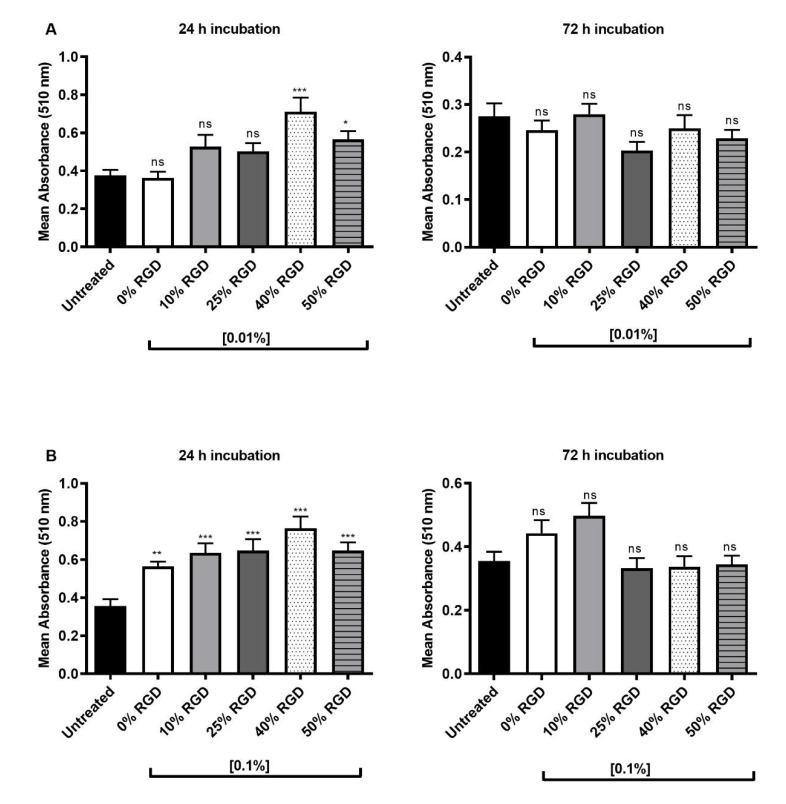
Biocompatibility studies of Stro-1 DPSCs with 0.01 and 0.1% *w*/*v* soluble forms of multicomponent peptides (0–50% RGD), as determined by the MTT assay. (**A**) Treatment of Stro-1 DPSCs with peptide concentrations of 0.01% *w*/*v* for 24 and 72 h. (**B**) Treatment of Stro-1 DPSCs with peptide concentrations of 0.1% *w*/*v* for 24 and 72 h. All comparisons were made with the untreated control. Data are from three experiments, with at least three replicates per experiment. Kruskal–Wallis test with Dunn’s correction for multiple comparison testing. ns: non-significant; (*p* > 0.05), *: *p ≤* 0.05, **: *p ≤* 0.01, ***: *p ≤* 0.001.

The biocompatibility of the hydrogel peptides in solution form were initially screened against a sub-population of Stro-1-positive DPSCs using standard 2D MTT assays. The results show that for 0.01% *w*/*v* peptides in solution form, only the 40% and 50% RGD hydrogel peptides significantly enhanced cellular metabolic activity at 24 h, as measured by the MTT assay (Figure 1A). At 72 h, no significant differences from untreated controls were observed (Figure 1A). When higher concentrations (0.1% *w*/*v*) of the peptides were tested in solution form, all peptide hydrogels showed significantly enhanced metabolic activity over controls at 24 h. No significant differences were observed after 72 h treatment, although the 0% and 10% RGD hydrogel peptide treatments remained higher than the controls (Figure 1B).

**Figure 2:**
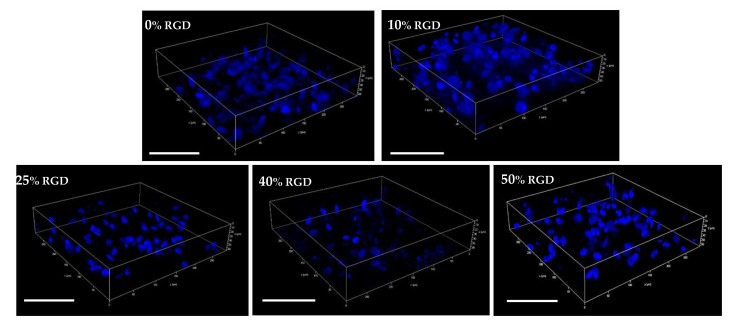
Confocal microscopy of encapsulated DPSCs (24 h) in 0% to 50% RGD hydrogels. Images show that cells were retained in 3D within all hydrogels. Cells were fixed and permeabilised within the hydrogel before staining of the nuclei of encapsulated DPSCs with DAPI. Images were captured using an SP8 Confocal Microscope. Scale bar: 100 µm.

**Figure 3:**
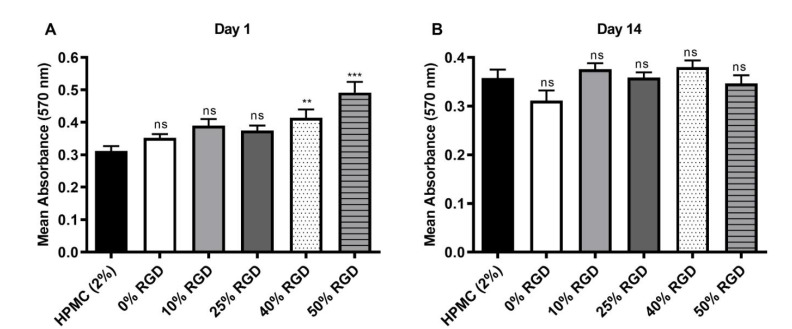
Modified MTT assay (for 3D culture) following encapsulation of DPSCs in HPMC hydrogel and 0–50% RGD assembled hydrogels at (**A**) day 1 and (**B**) day 14. All comparisons were made with the HPMC control. Data are from three experiments, with at least three replicates per experiment. Kruskal–Wallis test with Dunn’s correction for multiple comparison testing. ns: non-significant (*p* > 0.05), **: *p ≤* 0.01, ***: *p ≤* 0.001.

Having established biocompatibility with a sub-population of Stro-1-positive DPSCs, we then encapsulated the unsorted population of DPSCs in 3D hydrogels and showed by confocal microscopy (Figure 2) that they were maintained in 3D within all the hydrogels, following encapsulation. We also determined the biocompatibility of DPSCs with assembled hydrogels using a 3D MTT assay and showed that the 40 and 50% RGD peptides significantly enhanced cellular metabolic activity after 1 day (Figure 3A), but no significant differences from untreated controls were observed after 14 days in hydrogel culture (Figure 3B). Since biocompatibility over an extended time period is important for potential regenerative therapeutics, the 10% RGD peptide hydrogels were selected for further study, on the basis of their superior (although non-significant) biocompatibility at 72 h (Figure 1B).

### 3.2. Secretome of DPSCs in 2D versus 3D Culture

**Figure 4:**
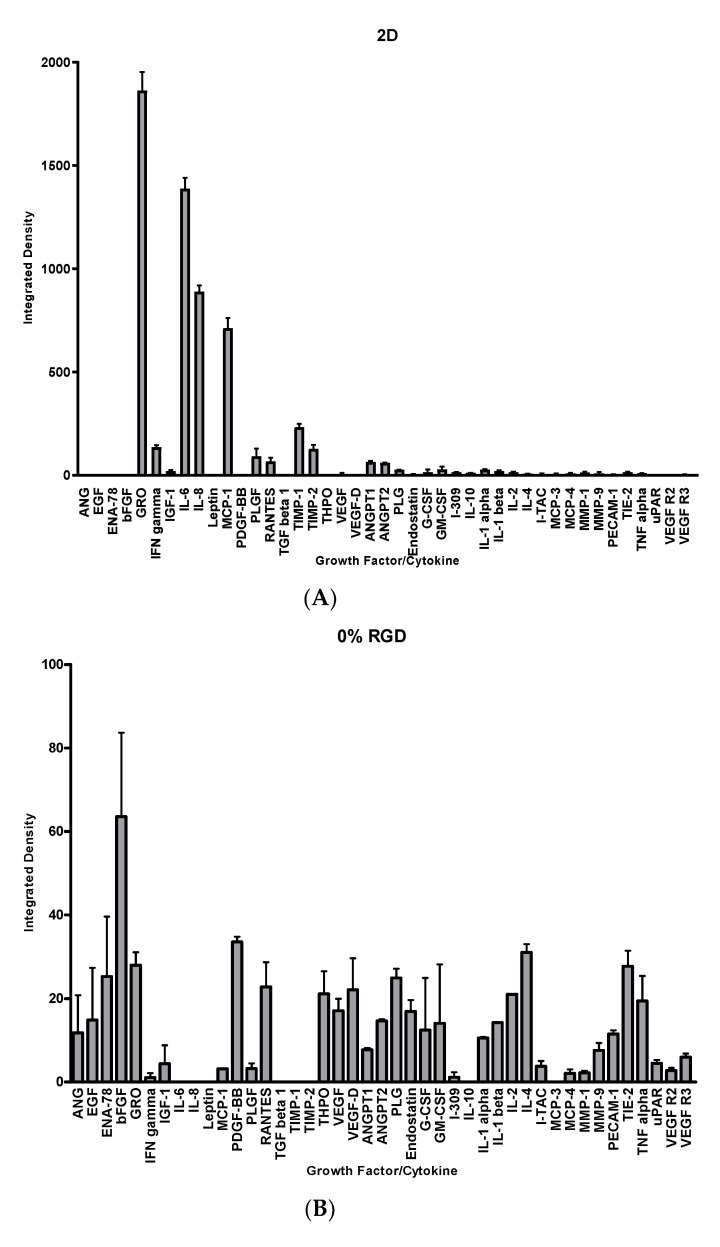

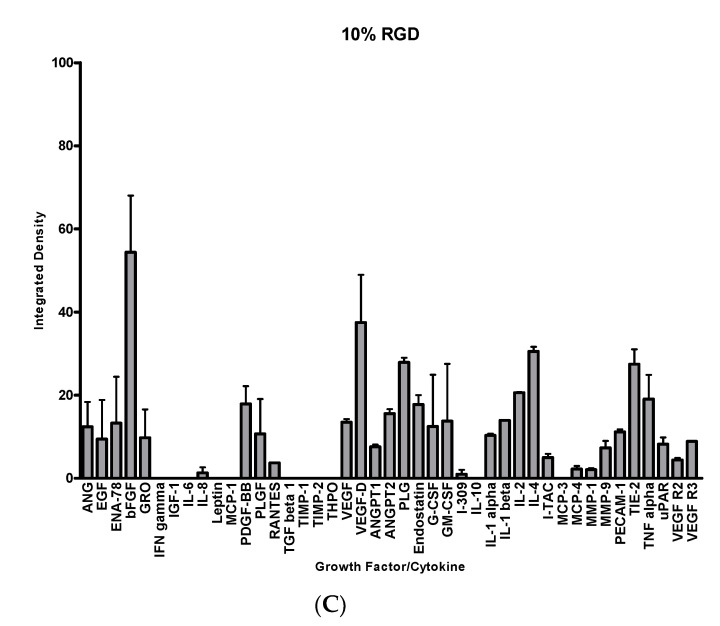
Growth factors/cytokine expression in conditioned media from DPSCs in (**A**) 2D culture or following 14-day encapsulation in (**B**) 0% RGD or (**C**) 10% RGD hydrogels. Abbreviations: angiogenin (ANG); epidermal growth factor (EGF); epithelial neutrophil-activating peptide 78 (ENA-78); basic fibroblast growth factor (bFGF); growth-regulated alpha protein (GRO-*α*); interferon gamma (IFN-*γ*); insulin-like growth factor 1 (IGF-1); interleukin-6 (IL-6); interleukin-8 (IL-8); leptin; monocyte chemoattractant protein-1 (MCP-1); platelet-derived growth factor BB (PGDF-BB); placental growth factor (PLGF); regulated upon activation normal T cell expressed and secreted (RANTES); transforming growth factor-beta 1 (TGF-*β*1); tissue inhibitor of matrix metalloproteinases-1 (TIMP-1); tissue inhibitor of matrix metalloproteinases-2 (TIMP-2); thrombopoietin (THPO); vascular endothelial growth factor (VEGF); vascular endothelial growth factor- D (VEGF-D); angiopoietin-1 (ANGPT1); angiopoietin-2 (ANGPT2); plasminogen (PLG); endostatin; granulocyte colony stimulating factor (G-CSF); granulocyte-macrophage colony-stimulating factor (GM-CSF); T lymphocyte-secreted protein I-309 (I-309); interleukin-10 (IL-10); interleukin-1 alpha (IL-1*α*); interleukin-1 beta (IL-1*β*); interleukin-2 (IL-2); interleukin-4 (IL-4); interferon-inducible T cell alpha chemoattractant (I-TAC); monocyte chemoattractant protein-3 (MCP-3); monocyte chemoattractant protein-4 (MCP-4); matrix metalloproteinase-1 (MMP-1); matrix metalloproteinase-9 (MMP-9); platelet and endothelial cell adhesion molecule-1 (PECAM-1); tyrosine kinase with immunoglobulin-like and EGF-like domains (TIE-2); tumour necrosis factor alpha (TNF*α*); urokinase plasminogen activator surface receptor (uPAR); vascular endothelial growth factor receptor 2 (VEGF R2); vascular endothelial growth factor receptor 3 (VEGF R3).

A total of 43 growth factors and cytokines were measured in the conditioned media from DPSCs grown in a standard 2D culture versus a 3D hydrogel culture (0% RGD or 10% RGD) using an angiogenic array. In the 2D culture, growth-regulated alpha protein (GRO-*α*), interleukin-6 (IL-6), IL-8 and monocyte chemoattractant protein-1 (MCP-1) were detected most intensely (Figure 4A). However, in the 3D culture (0% or 10% RGD hydrogel), GRO-*α* and MCP-1 were not as abundant, and IL-6 and IL-8 were barely detectable. On the contrary, a number of growth factors and cytokines, such as angiogenin (ANG), epidermal growth factor (EGF), epithelial neutrophil-activating peptide 78 (ENA-78), platelet-derived growth factor BB (PDGF-BB) and vascular endothelial growth factor- D (VEGF-D), were detectable in the 3D culture (0% RGD and 10% RGD) (Figure 4B,C). Interestingly, there was some variability in growth factors and cytokine expression, depending on the presence or absence of RGD. For example, PDGF-BB levels were higher in the 0% RGD versus 10% RGD culture, and thrombopoietin (THPO) was not detectable in the 10% RGD culture at all. On the other hand, VEGF-D levels were higher in the 10% RGD versus 0% RGD culture (Figure 4B,C).

### 3.3. Antibacterial Properties of Soluble Peptides and Assembled Peptide Hydrogels

**Figure 5:**
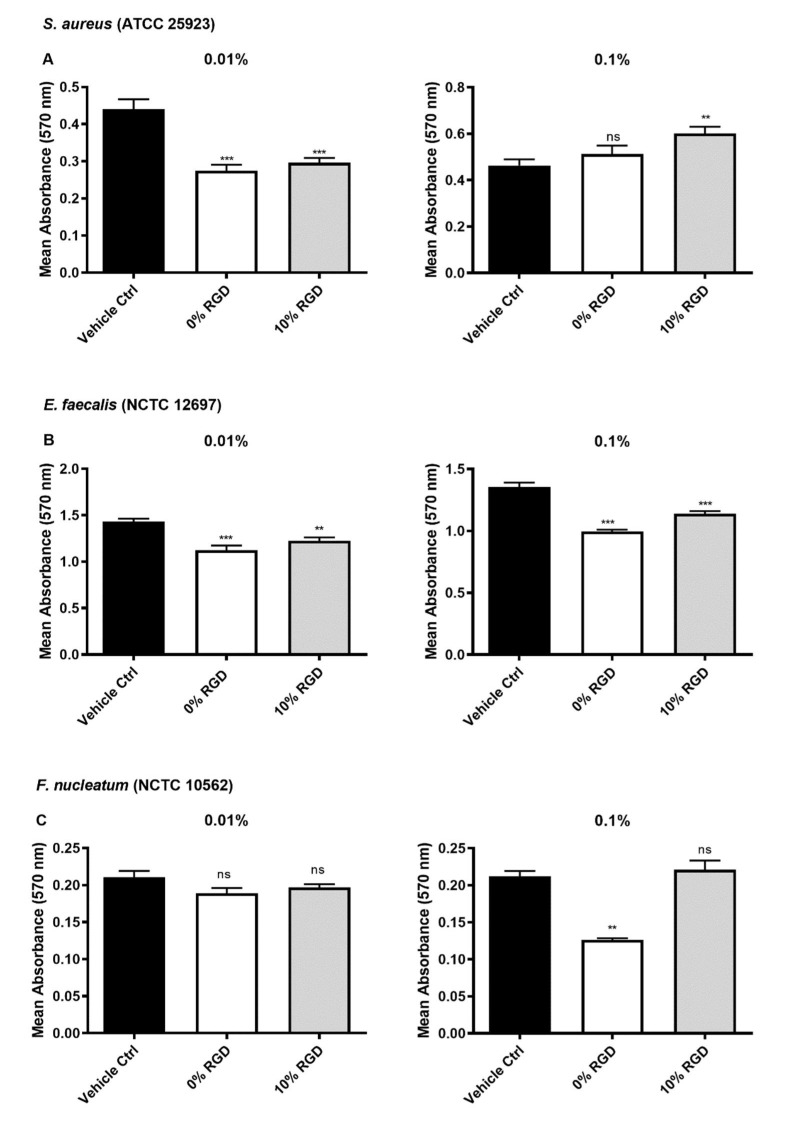
Biofilm inhibition by 0% RGD and 10% RGD soluble peptides, as determined by the crystal violet assay. (**A**) *S. aureus*, (**B**) *E. faecalis* and (**C**) *F. nucleatum* biofilm biomass following 24 h incubation with 0.01% and 0.01% *w*/*v* peptides. All comparisons were made with vehicle controls. Data are from three experiments, with at least three replicates per experiment. *S. aureus* and *E. faecalis* data analysed by one-way ANOVA with Dunnett’s correction for multiple comparison testing. *F. nucleatum* data analysed by a Kruskal–Wallis test with Dunn’s correction for multiple comparison testing. ns: non-significant (*p* > 0.05), **: *p ≤* 0.01, ***: *p ≤* 0.001.

In order to investigate the antibacterial properties of the peptide hydrogels (+/− RGD), it was important to determine whether low concentrations of the peptides (representing soluble peptides that could potentially diffuse from the hydrogel surface) had antibacterial activity against biofilms of oral pathogens. There was a significant reduction in the biomass of *S. aureus* biofilms following treatment with the 0% RGD and 10% RGD peptides at a concentration of 0.01% *w*/*v*, but not at a concentration of 0.1% *w*/*v* (Figure 5A). The biofilm biomass of *E. faecalis* biofilms was significantly reduced following treatment with the 0% RGD and 10% RGD peptides, at both concentrations tested (0.01% and 0.1% *w*/*v*). When *F. nucleatum* biofilms were treated, only the 0% RGD hydrogel peptides were shown to significantly reduce biofilm biomass at a concentration of 0.1% *w*/*v* (Figure 5C).

**Figure 6:**
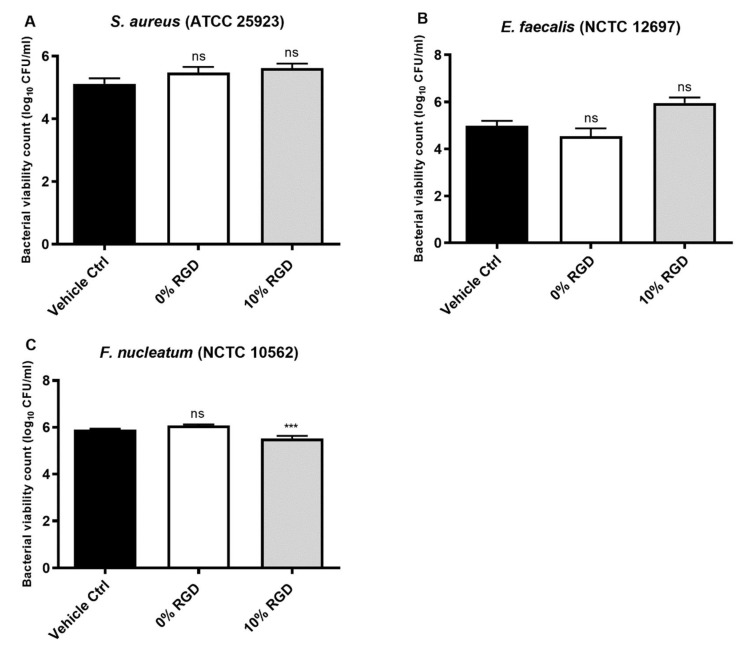
Bacterial viability counts (Log_10_ CFU/mL) following 24 h growth on the surface of 0% RGD or 10% RGD assembled hydrogels. (**A**) *S. aureus*, (**B**) *E. faecalis* and (**C**) *F. nucleatum*. All comparisons were made with untreated controls. Data are from three experiments, with at least three replicates per experiment. *S. aureus* and *F. nucleatum* data analysed by one-way ANOVA with Dunnett’s correction for multiple comparison testing. *E. faecalis* data analysed by a Kruskal–Wallis test with Dunn’s correction for multiple comparison testing. ns: non-significant (*p* > 0.05), ***: *p ≤* 0.001.

The antibacterial activity of the assembled hydrogels was then tested against *S. aureus*, *E. faecalis* and *F. nucleatum*, using a CFU viable count assay. After 24 h bacterial growth on the hydrogel surface, there was no significant change in the number of CFU of *S. aureus* or *E. faecalis*, but *F. nucleatum* numbers were significantly reduced by the 10% RGD assembled hydrogel (Figure 6).

## 4. Discussion

In the development of hydrogels that are biocompatible with a variety of human stem/stromal cell types, a popular approach has been to employ functional fragments of ECM proteins, including the cell adhesion motif RGD [27]. Whilst the majority of studies focus on the biocompatibility [28] or cell attachment properties of RGD, some researchers have also investigated the addition of nanoparticles to RGD-containing hydrogels to generate novel antimicrobial properties [29]. In the current study, we examined the biocompatibility of peptide hydrogels synthesised without (0%) or with (10–50%) of the RGD cell adhesion motif. We also investigated the angiogenic secretome of DPSCs cultured in 2D versus 0% or 10% RGD in 3D hydrogel culture. In addition, we investigated the antimicrobial properties of the peptide hydrogels (0% and 10% RGD) against three oral pathogens.

Our results show that the hydrogels were biocompatible with both Stro-1-positive DPSCs and unsorted DPSCs. Stro-1 sorting of DPSCs is often undertaken to select a sub-population of DPSCs of mesenchymal origin, with the propensity for odontogenic differentiation [30]. The 10% RGD hydrogel showed more favourable biocompatibility at 72 h against Stro-1-positive cells. However, if hydrogels are to be translated for therapeutic use, then it is also important that their biocompatibility against unsorted DPSCs is studied in 3D cultures. Using confocal microscopy, we showed that all the hydrogels tested were capable of retaining DPSCs in 3D, and also that all hydrogels displayed similar biocompatibilities with DPSCs after 14 days in 3D culture. It is worth noting that we used encapsulated cells for 3D culture, since cells are often grown on top of flat hydrogels/hydrogel surfaces as simplified methods for biocompatibility studies [27,31].

The selection of the 10% RGD hydrogel, over higher % RGD hydrogels for further study, is in line with previous work, which suggests that too many RGD anchors may hinder cellular activity within the scaffold [32]. The hydrogels used in this study contained a homogenous mixture of 10% RGD functionalized peptides and 90% non-RGD peptides. Previous work has shown that there are subtle differences between hydrogels containing homogenous RGD versus clustered RGD. Clustering of RGD has been reported to facilitate cell spreading/elongation within the hydrogel [32,33], but may not have the initial cell adhesion characteristics of homogenous RGD [32].

With a view to determining the effect of 3D hydrogel culture on the DPSC secretome, we investigated angiogenic growth factor/cytokine release from DPSCs in 2D versus 3D (0% or 10% RGD) hydrogel culture. The release of angiogenic factors plays an important role in repair and regeneration in the dental pulp, and thus the synthesis and release of such factors from cells within the hydrogel could provide the microenvironment needed for angiogenesis, as an alternative to supplementation by exogenous angiogenic factors [34,35]. Analysis of the DPSC secretome showed that culture in 3D resulted in the decreased expression of pro-inflammatory factors such as IL-6 and IL-8, along with an increase in the expression of pro-angiogenic factors such as ANG, EGF, ENA-78, PDGF-BB, and VEGF-D. When 0% RGD and 10% RGD 3D hydrogel cultures were compared, some factors showed decreased levels in 10% RGD (PDGF-BB and THPO), whereas VEGF-D levels were higher in the 10% RGD versus 0% RGD culture. Interestingly, it has previously been hypothesised that RGD hydrogels activate the αvβ3 integrin on cells to cause heightened sensitivity to the presence of VEGF within the microenvironment [36]. This effect, together with the increased levels of VEGF in 10% RGD hydrogels, could enhance angiogenesis.

Hydrogels are currently being designed to incorporate functional antimicrobial components; examples include the incorporation of silver moieties [29] or antibiotics [37] (reviewed in [38]). However, although many peptides have inherent antimicrobial activity, peptide hydrogels have not conventionally been assessed for their inherent antimicrobial effects. We initially tested the antibiofilm effects of low concentrations of soluble peptide components of the hydrogels, as it has previously been debated that antimicrobial activity is not exclusive to the process of gelation and that the presence of soluble peptides around the hydrogel surface could also have a role in determining antimicrobial efficacy [14,24]. Thus, peptides in soluble form may be present on the hydrogel surface [13,14,24] and could contribute to antimicrobial activity. Solutions of 0.01% *w*/*v* peptides (0% RGD and 10% RGD) were effective against *S. aureus* and *E. faecalis* biofilms. A 0.1% *w*/*v* solution of 0% RGD was required for efficacy against *F*. *nucleatum*, but the 10% RGD culture had no significant antibiofilm effect. The exact mechanisms of action of the peptides in solution on the hydrogel surface have not been investigated, but it is likely that they cause membrane disruption in a similar manner to that reported for naturally occurring antimicrobial peptides [39]. Many antimicrobial peptides achieve their antimicrobial effects by disrupting the bacterial lipid bilayer, leading to the leakage of cell contents and ultimately bacterial cell death [40]. The efficacy of various antimicrobial peptides against different bacteria has been reported to depend on the membrane lipid composition [40]. Hence, the selectivity of antimicrobial peptides against the different bacterial strains studied in the present work could be attributed to differences in bacterial membrane lipid composition [41]. It has also been reported that additional protection against antimicrobials is offered by the outer membrane of Gram-negative bacteria [42]. The presence of the outer membrane could contribute to the tendency of *F. nucleatum*, particularly in biofilm form, to be more resistant to treatment with 0.01% *w*/*v* soluble peptides, as observed in the current study. It is important to note that the antibiofilm activity is not only related to the ability of the peptide to interact with and disrupt the bacterial membrane, but also on its ability to penetrate the biofilm matrix [43]. Biofilms are widely recognised to be recalcitrant to treatment with antibiotics [44] or antimicrobial peptides [45], emphasising the need for further work in this research field [46].

In addition to investigating the antimicrobial activity of soluble peptides, we also investigated whether the assembled hydrogels had antimicrobial properties [47]. It is worth noting that the peptides in solution displaying anti-biofilm efficacy did not directly translate to antibacterial efficacy in assembled hydrogel form, as determined by the CFU assay and vice versa. There are numerous factors that could contribute to this apparent disparity. The process of self-assembly has previously been reported to influence antimicrobial properties [47]. It is thought that a combination of factors, including changes in secondary structure and charge distribution, particularly the surface charge and the ratio of charge to surface area, will occur in peptide assembly, thereby influencing peptide–bacterial interactions [47]. It was interesting to note that one assembled hydrogel, namely 10% RGD, displayed antimicrobial activity, but only against *F. nucleatum*. Since the RGD motif contains one positively charged amino acid (R), one uncharged amino acid (G) and one negatively charged amino acid (D), it is unlikely that RGD incorporation affected the overall charge of the 10% RGD versus the 0% RGD peptide hydrogel. However, as discussed above, it is possible that self-assembly of the peptides may have altered the charge distribution in the assembled hydrogel, facilitating antimicrobial efficacy that was not observed with the soluble peptides. Comparisons of the efficacies of the soluble and assembled hydrogel forms of the peptides are, however, difficult, because their antimicrobial activities were tested against biofilms, or bacteria seeded on the hydrogel surface, respectively. Moreover, it is likely that both the soluble and assembled hydrogel forms could contribute to antimicrobial activity in vivo. Thus, any reduction in bacterial burden by hydrogel components, combined with the host immune response, could contribute to successful resolution of infection.

In conclusion, peptide hydrogels (+/− RGD) are biocompatible with DPSCs and have antibacterial activity against oral pathogens. In addition, the hydrogels were shown to influence the secretome of encapsulated cells. Replicating the subtle concentration gradients and temporal changes of exogenously applied growth factors for tissue engineering purposes is a complex process, associated with significant regulatory issues. However, the ability to engineer hydrogel scaffolds so that the cells encapsulated within them produce their own growth factors, as well as exhibiting inherent antibacterial and biocompatible characteristics, could greatly improve their clinical efficacy.

## Figures and Tables

**Table 1:** Composition of standard Biogelx hydrogel (Biogelx-S) (0% RGD) and custom synthesized RGD-containing hydrogels.

Hydrogel	Components (% moles)		
	Fmoc-FF (%)	Fmoc-S (%)	Fmoc-RGD (%)
0% RGD	50	50	0
10% RGD	50	40	10
25% RGD	50	25	25
40% RGD	50	10	40
50% RGD	50	0	50

## Data Availability

Data are available from the corresponding author upon reasonable request.
